# To die or not to die? Lessons from lesion mimic mutants

**DOI:** 10.3389/fpls.2015.00024

**Published:** 2015-01-30

**Authors:** Quentin Bruggeman, Cécile Raynaud, Moussa Benhamed, Marianne Delarue

**Affiliations:** ^1^Institut de Biologie des Plantes, UMR CNRS 8618, Université Paris-Sud, Saclay Plant SciencesOrsay, France; ^2^Division of Biological and Environmental Sciences and Engineering, Center for Desert Agriculture, King Abdullah University of Science and TechnologyThuwal, Saudi Arabia

**Keywords:** plant, programmed cell death, lesion mimic mutants, genetics approaches, immunity responses

## Abstract

Programmed cell death (PCD) is a ubiquitous genetically regulated process consisting in an activation of finely controlled signaling pathways that lead to cellular suicide. Although some aspects of PCD control appear evolutionary conserved between plants, animals and fungi, the extent of conservation remains controversial. Over the last decades, identification and characterization of several lesion mimic mutants (LMM) has been a powerful tool in the quest to unravel PCD pathways in plants. Thanks to progress in molecular genetics, mutations causing the phenotype of a large number of LMM and their related suppressors were mapped, and the identification of the mutated genes shed light on major pathways in the onset of plant PCD such as (i) the involvements of chloroplasts and light energy, (ii) the roles of sphingolipids and fatty acids, (iii) a signal perception at the plasma membrane that requires efficient membrane trafficking, (iv) secondary messengers such as ion fluxes and ROS and (v) the control of gene expression as the last integrator of the signaling pathways.

## Introduction

The decision whether a cell should live or die is fundamental to the survival of multicellular organisms. Programmed Cell Death (PCD), a genetically regulated cellular suicide, has been described in all multicellular organisms and recent studies even suggest that apoptosis-like processes could occur in bacteria (Hakansson et al., 2011; Dwyer et al., 2012). In plants, PCD is involved both in processes required for normal development and to face biotic or abiotic stress (Pennell and Lamb, 1997; Williams and Dickman, 2008). From a cellular perspective, plant PCD can be classified as (i) autolytic PCD, characterized by the formation of large lytic vacuoles and rapid clearance of the cytoplasm due to tonoplast rupture and the release of hydrolases, and (ii) non-autolytic PCD which lacks rapid clearance of the cytoplasm (Van Doorn, 2011). Autolytic PCD occurs mainly during plant development whereas non-autolytic PCD is generally observed in response to stress, for example during the hypersensitive response (HR) induced by pathogens (Van Doorn, 2011). HR is one of the best characterized PCD (Coll et al., 2011) and phytohormones such as salicylic acid (SA), jasmonic acid (JA) or ethylene (ETH), as well as reactive oxygen species such as hydrogen peroxyde (H_2_O_2_), singlet oxygen (^1^O_2_) or superoxide anion (O^−^_2_), are key players for HR development and regulation (Coll et al., 2011). However, our understanding of the cellular events leading to cell death in plants remains largely incomplete.

By contrast, in mammalian cells, the molecular bases of PCD are well described. The main regulators of PCD in animals belong to the Bcl-2 family of proteins composed of pro (Bid, Bad, Bak, and Bax) and anti-apoptotic proteins (Bcl-2 and Bcl-xL) (Youle and Strasser, 2008). Under normal conditions, mitochondrial outer membrane integrity is maintained through a balance between these pro and anti-apoptotic proteins. Perception of a pro-death signal can activate factors such as BAX and BAK (for Bcl2-ASSOCIATED X and Bcl2-HOMOLOGS ANTAGONIST/KILLER) that allow the release of cytochrome *c* (cyt. *c*) from mitochondria. Once in the cytoplasm, cyt. *c* triggers a chain reaction by activating caspases (cysteine-aspartyl proteases), which target various proteins, leading to the activation of several hydrolases and thus to the degradation of most cellular macromolecules (Marino et al., 2014). Based on sequence homology, some caspase-like proteins with protease activities have been identified in plants, such as metacaspases (Tsiatsiani et al., 2011), but this approach was not very fruitful, due to the absence of clear sequence conservation between animals and plants, and it thus remains unclear whether the pathways described in animal cells are conserved in plants. In particular, plant genomes appear to lack Bcl-2 family homologs. Nevertheless, expression of the murine BAX protein in Tobacco or *Arabidopsis* is sufficient to promote cell death (Lacomme and Santa Cruz, 1999; Kawai-Yamada et al., 2001), and release of cyt. *c* during plant PCD has been documented (Colombatti et al., 2014), indicating that mitochondria also play a central role during plant PCD. In addition, the BAX-INHIBITOR 1 (BI-1) PCD antagonist is conserved in plants where it can prevent cell death induced by ectopic expression of BAX (Kawai-Yamada et al., 2001), and control cell death progression during pathogen attacks (Watanabe and Lam, 2006). Hence, BI-1 regulates cell death in plants, but data available so far suggest that the molecular mechanisms underlying its action and its partners are not conserved between plants and animals and that sequence similarity searches are thus probably not the best suited to decipher PCD control in plants. Others strong candidates that may function as caspase-like executioners of plant PCD are vacuolar processing enzymes (VPE). Even though they display little sequence homology to animal caspases, they were found to have YVADase (caspase-1 like) activity, and VPE deficiency was shown to prevent virus-induced HR cell death in tobacco (Hatsugai et al., 2004). δVPE was also reported to be involved in the cell death associated with early seed development (Nakaune et al., 2005).

Extensive progress in our understanding of plant PCD in response to stress came from forward genetic approaches and the identification of many mutants displaying spontaneous HR-like cell death, on leaves: the so called lesion-mimic mutants (LMM).

Since about 30 years, at least 60 of these mutants have been isolated in maize (Hoisington et al., 1982), rice (Takahashi et al., 1999), barley (Wolter et al., 1993) and Arabidopsis (Lorrain et al., 2003; Moeder and Yoshioka, 2008). The combined efforts of laboratories and advances in genome annotation of model species allowed mapping of the corresponding mutations in a large number of LMM and the identification of the mutated genes. Table 1 lists the 49 LMM for which the mutated genes have been identified, and summarizes the phenotypes of the mutants, as well as the putative underlying mechanisms causing PCD. Because most mutations present in these LMM are recessive, causing a loss of protein function, this first approach led mainly to the identification of inhibitors of cell death. In order to find positive regulators of PCD, second site mutageneses have been performed by several laboratories. These suppressors are listed in Table 2, indicating which LMM is suppressed, the molecular function of the gene product, and the possible mechanism involved in the suppression of PCD.

**Table 1:** **Lesion mimic mutants**.

**Mutant name**	**Gene ID**	**Gene product**	**PCD-related phenotypes**	**Possible mechanisms involved**	**References**
*acd1*	At3g44880	Pheide a oxygenase involved in chlorophyll catabolic process	Lesions on older leaves, or when plants are submitted to a dark-light transfer	Accumulation of photoreactive pheide a	Greenberg and Ausubel, 1993; Gray et al., 1997; Tanaka et al., 2003
*lls1* (Zm)					
*acd2*	At4g37000	Red chlrorophyll catabolite reductase involved in chlorophyll catabolic process	Lesions on older leaves	Accumulation of red chlorophyll catabolites that causes singlet oxygen release	Mach et al., 2001; Yao and Greenberg, 2006; Pruzinska et al., 2007
*acd5*	At5g51290	Ceramide kinase	Small restricted spontaneous lesions on leaves 5 weeks after planting	Accumulation of non-phosphorylated C2 ceramide	Greenberg et al., 2000; Liang et al., 2003; Bi et al., 2011
*acd6* (gof)	At4g14400	Transmembrane protein with ankyrin domain	Yellowing leaves with cell death patches 2 weeks after planting	Constitutive activation of defense responses	Rate et al., 1999; Lu et al., 2003
*acd11*	At2g34690	Protein that transfers Ceramide-1-Phosphate between membrane	Chlorosis that can engulfed the rosette	Ceramide and phytoceramide accumulation	Brodersen et al., 2002; Petersen et al., 2008; Simanshu et al., 2014
*agd2*	At4g33680	Chloroplastic aminotransferase	Spotted necrotic lesions	Unknown	Rate and Greenberg, 2001; Song et al., 2004
*bir1*	At5g48380	Receptor-like kinase belonging to the LRRX group	Lesions in cotyledons and true leaves	Activation of multiple defense signaling pathways	Gao et al., 2009
*cad1*	At1g29690	Protein containing a MACPF domain	HR-like lesions on leaves	Unknown	Morita-Yamamuro et al., 2005
*camta3*/*sr1*	At2g22300	Calmodulin-binding transcription factor	Chlorotic lesions on rosette leaves	Overexpression of immune regulator such as *EDS1* and *EIN3*	Yang and Poovaiah, 2002; Galon et al., 2008; Du et al., 2009
*er1* (Nt)					
*cat2*	At4g35090	Dismutation of H_2_O_2_	Necrotic lesions in Long-Days conditions	Intracellular oxidative stress coupled with decreased of Myo-inositol content	Vandenabeele et al., 2004; Queval et al., 2007; Chaouch and Noctor, 2010
*cea62* (Os)	Os02g0110200	hydroperoxyde lyase	Brown lesion spots over the entire leaf surface	Probably due to constitutive induction of JA signaling	Liu et al., 2012
*chs2* (gof)	At4g16860	Encodes the TIR-NBS-LRR protein named RPP4	Lesions and bleaching when transfer in low temperature conditions	Conditional activation of defense responses	Huang et al., 2010
*cpn1/bon1*	At5g61900	Calcium-dependent phospholipid binding protein	Lesions appear on leaves in low humidity and/or low temperature conditions	Overexpression of SNC1 is necessary to induce lesions	Jambunathan et al., 2001; Jambunathan and McNellis, 2003; Yang and Hua, 2004
*cpr5*	At5g64930	Transmembrane protein with unknown molecular function	Spontaneous lesions and precocious senescence	Unknown	Bowling et al., 1997; Kirik et al., 2001; Yoshida et al., 2002
*cpr22* (gof)	At2g46450 and At2g46440	Cyclic-gated ion channel for Ca^2+^	Chlorotic lesions, then plants die within 14 days after planting	The chimeric protein CNGC11/12 created by *cpr22* deletion constitutively activate defense signaling	Yoshioka et al., 2001, 2006; Urquhart et al., 2007
*cslf6* (Os)	Os08g06380	Cellulose synthase-like involved in mixed-linkage glucan biosynthesis	Spontaneous, discrete, necrotic lesions in flag and old leaves	Probably due to high decrease in mixed-linkage glucan content	Vega-Sanchez et al., 2012
*dnd1*	At5g15410	Cyclic-gated ion channel for Ca^2+^	Small necrotic lesions but also a decrease in HR induction	Unknown	Yu et al., 1998; Clough et al., 2000; Ali et al., 2007
*dnd2*/*hlm1*	At5g54250	Cyclic-gated ion channel for K^+^, Na^+^ and/or Ca^2+^	Spontaneus lesions but also a decrease in HR induction	Unknown	Balague et al., 2003; Jurkowski et al., 2004; Rostoks et al., 2006
*nec1* (Hv)					
*edr1*	At1g08720	Map Kinase Kinase Kinase	Necrotic lesions in the case of infection with the powdery mildew	Derepression of a MAP kinase cascade in *edr1* seems to be responsible of the cell death phenotype	Frye and Innes, 1998; Frye et al., 2001; Zhao et al., 2014
*edr2*	At4g19040	a PH and STAR domain containing protein	Necrotic lesions in the case of infection with the powdery mildew	Unknown	Tang et al., 2005
*edr3*	At3g60190	Dynamin-related protein with a putative role in Golgi traffic	Necrotic lesions in the case of infection with the powdery mildew	Unknown	Hong et al., 2003; Tang et al., 2006
*erh1*	At2g37940	Inositol phosphorylceramide synthase	Spontaneous lesions in a Col-0 genetic background expressing the resistance gene RPW8	Ceramide accumulation	Wang et al., 2008
*erh1* (Os)					
*exo70b1*	At5g58430	Exocyst subunit EXO70 family protein B1 with a role in autophagy	HR-like lesions	Unknown	Kulich et al., 2013; Stegmann et al., 2013
*flu*	At3g14110	Protein with Coiled-coil TPR domain regulating chlorophyll biosynthesis	Lesions on mature leaves, complete bleaching of seedling after a dark to light transfer	Accumulation of Pchlide in the dark which then generate singlet oxygen in the light condition	Meskauskiene et al., 2001; Op Den Camp et al., 2003; Przybyla et al., 2008; Khandal et al., 2009
*tigrina*-*d*.*12* (Hv)					
*fzl*	At1g03160	Membrane remodeling GTPase localized in chloroplast enveloppe	Chlorotic lesions on rosettes leaves in Ler ecotype but not in Col-0	Probably caused by multiple chloroplasts anomalies observed in *fzl*	Gao et al., 2006; Landoni et al., 2013
*len1*	At1g55490	Chaperonin 60β localized in chloroplast	Spontaneous lesions only in Short-days but not in Long-days condtions	Unknown	Ishikawa et al., 2003; Ishikawa, 2005
*les22* (Zm)	GRMZm2G044074	Uroporphyrynogen decarboxylase (Chlorophyll biosynthesis)	HR-like necrotic lesions	Probably due to accumulation of uroporphyrine	Hu et al., 1998
*loh1*	At3g25540	Ceramide synthase	lesions after a long growth in Short-days condition	Accumulation of free trihydroxy sphingoid bases or ceramide species with C16 fatty acids	Ternes et al., 2011
*lrgB*	At1g32080	Intermembrane protein localized in chloroplast envelop inner membrane	Chlorotic lesions on true leaves of juveniles plants	Unknown	Yamaguchi et al., 2012; Yang et al., 2012
*lin2*	At1g03475	Coproporphyrinogen III oxidase involved in chlorophyll biosynthesis	Spontaneous lesions formation on young leaves	Probable accumulation of photosensitizing tetrapyrrole intermediates	Ishikawa et al., 2001; Sun et al., 2011; Guo et al., 2013
*Rlin1* (Os)					
*lms* (Os)	Os02g0639000	Protein with a CTD phosphatase domain and two dsRBM motifs	Reddish-brown lesions on leaves and rapid senescence after flowering	Unknown	Undan et al., 2012
*lsd1*	At4g20380	Protein containing three zinc finger domains	Spontaneous and runaway cell death on leaves when plants are transferred from Short-days to Long-days conditions or submitted to a highlight stress	Uncontrolled oxidative stress (anion superoxide and oxydized plastoquinone pool) that is the consequence of misregulation of numerous genes	Jabs et al., 1996; Dietrich et al., 1997; Kliebenstein et al., 1999; Muhlenbock et al., 2008
*lsd1* (Os)					
*mips1*	At4g39800	Myo-inositol-1-phosphate synthase which catalyze the first reaction of Myo-inositol biosynthesis	Necrotic lesions appear on rosette leaves after a transfer from Short-days to Long-days conditions	Not clearly determined but a ceramide accumulation can be observed	Meng et al., 2009; Donahue et al., 2010; Luo et al., 2011
*mkk1/mkk2*	At4g26070, At4g29810	Two Map kinase kinase which act to negatively regulate plant defenses	Reduced growth, lesions that engulfed the whole seedling and lead to lethality	Constitutive activation of defense responses	Ichimura et al., 1998; Gao et al., 2008; Qiu et al., 2008
*mpk4*	At4g01370	Map kinase which acts downstream to MKK1/MKK2 to negatively regulate plant defenses	Reduced growth, small spotted lesions	Constitutive activation of defense responses	Petersen et al., 2000; Su et al., 2007; Gao et al., 2008; Liu et al., 2011
*mpk4* (Gm)					
*nsl1*	At1g28380	Protein containing a MACPF domain	Spotted necrotic lesions	Unknown	Noutoshi et al., 2006
*pub13*	At3g46510, Os12g0570000	Protein with E3 ubiquitin ligase activity	Chlorosis and spotted lesions on leaves in LD conditions	Constitutive activation of defense responses	Zeng et al., 2004; Li et al., 2012
*spl11* (Os)					
*rug1*	At5g08280	Porphobilinogen deaminase involved in chlorophyll biosynthesis	Spontaneous lesions on leaves	Probably due to accumulation of porphobilinogen	Quesada et al., 2013
*siz1*	At5g60410	SUMO E3 ligase	Spontaneous lesions on leaves and dwarfism	Constitutive activation of defense responses	Lee et al., 2007a; Miura et al., 2010
*slh1*	At5g45260	TIR-NBS-LRR with a WRKY domain	Necrotic lesions on rosette and cauline leaves in low humidity	Defect in DNA binding of the protein that conduces to Constitutive activation of defense responses	Noutoshi et al., 2005
*smg7*	At5g19400	Involved in Nonsense-Mediated RNA decay process	Necrotic lesions on rosette leaves and lethality (depending on allele)	Constitutive activation of defense responses	Riehs-Kearnan et al., 2012
*spl7* (Os)	Os05g0530400	Heat stress transcription factor	Small, reddish brown lesions over the whole surface of leaves	Unknown	Yamanouchi et al., 2002
*spl28* (Os)	Os01g0703600	Clathrin adaptator subunit (AP1M1) involved in post-golgi traffic	Spotted lesions on leaves	Unknown	Qiao et al., 2010
*ssi2*	At2g43710	Steroyl-ACP desaturase involved in fatty acid desaturation	HR-like lesions	Increase in 18:0 and decrease in 18:1 fatty acids, inducing, among other things, NO accumulation	Kachroo et al., 2001, 2008; Shah et al., 2001; Mandal et al., 2012
*ssi2* (Gm, Os)					
*ssi4* (gof)		Putative TIR-NBS-LRR protein	Chlorotic lesions suppressed by high humidity	Probably caused by constitutive activation of defense signaling	Shirano et al., 2002; Zhou et al., 2004
*syp121/syp122*	At3g11820, At3g52400	Syntaxins involved in vesicular trafficking	Dwarf phenotype and necrotic lesions	Constitutive activation of defense responses	Zhang et al., 2008
*upf1*	At5g47010	Involved in Nonsense-Mediated RNA decay process	Necrotic lesions on rosette leaves and lethality (depending on allele)	Constitutive activation of defense responses	Riehs-Kearnan et al., 2012
*vad1*	At1g02120	Membrane-associated protein with a GRAM domain	Vascular necrotic lesions	Unknown	Lorrain et al., 2004
*xth27*	At2g01850	Xyloglucan endotransglucosylase/hydrolase	Yellow necrotic spots associated with leaf maturation	Unknown	Matsui et al., 2005

Bo, Brassica oleracea; Gm, Glycine max; Hv, Hordeum vulgare (barley); Ta, Triticum aestivum; Zm, Zea mays; Nt, Nicotiana tabbacum; gof, the mutation in correspondings lmm is a Gain of function. In all other lmm, the mutation is recessive.

**Table 2:** **Suppressors of LMM**.

**Mutant**	**lmm supressed**	**Gene ID**	**Gene Product**	**Possible mechanism involved**	**References**
*act1*	*ssi2*	At1g32200	Chloroplastic glycerol-3-phosphate acyltransferase	*act1* mutant accumulates 18:1 fatty acid within the chloroplast, this could explain the suppression of *ssi2* cell death	Kachroo et al., 2003
*adr1*	*lsd1*	At1g33560	CC-NBS-LRR immune receptor	ADR1 is a positive regulator of SA signaling	Bonardi et al., 2011; Roberts et al., 2013
*adr1-l1*	*lsd1*	At4g33300	CC-NBS-LRR immune receptor	ADR1-L1 is a positive regulator of SA signaling	Bonardi et al., 2011; Roberts et al., 2013
*adr1-l2*	*lsd1*	At5g04720	CC-NBS-LRR immune receptor	ADR1-L2 is a positive regulator of SA signaling	Bonardi et al., 2011; Roberts et al., 2013
*edts5/ald1*	*edr2 agd2*	At2g13810	Aminotransferase homologs to AGD2	Unknown	Nie et al., 2011
*bzip10*	*lsd1*	At4g02640	BZIP transcription factor	In wild-type plants, LSD1 retain BZIP10 in cytoplasm. In *lsd1* mutants, BZIP10 translocate into nucleus to regulate expression of genes involved in *lsd1* cell death	Kaminaka et al., 2006
*caa39*	*flu*	At5g02820	Subunit of the topoisomerase VI complex (TOP6A)	TOP6A binds to promoter of genes regulated by ROS signaling.	Simkova et al., 2012
*cao*	*lsd1*	At5g50920	Chaperone necessary for the assembly of the photosystem II antenna	A fully functional photosystem II is necessary for lesion formation in *lsd1*	
*crt1*	*ssi4*	At4g36290	ATPase that interacts with diverse resistance proteins	Unknown	Kang et al., 2008
*crt3*	*bir1*	At1g08450	Calreticulin involved in the ER quality control	*crt3* and *erdj3b* are characterized by a decrease of SOBIR1 accumulation	Sun et al., 2014
*erdj3b*	*bir1*	At3g62600	J domain protein involved in the ER quality control		Sun et al., 2014
*ex1*	*flu*	At4g33630	Chloroplastic protein with unknown molecular fucntion	EX1 and EX2 act together to integrate ^1^O_2_signal from chloroplasts and subsequently induce signaling responses that induce cell death in *flu*	Wagner et al., 2004; Lee et al., 2007b; Kim et al., 2012
*ex2*	*flu*	At1g27510	Chloroplastic protein with unknown molecular fucntion		Lee et al., 2007b; Kim et al., 2012
*hpr1*	*edr1*	At5g09860	Component of the THO/transcrtiption export complex required for mRNA export	A fully functional mRNA export machinery is necessary for cell death in *edr1*	Pan et al., 2012
*laz1*	*acd11*	At4g38360	Protein with a domain of unknown function (DUF300)	Unknown	Malinovsky et al., 2010
*laz2*	*acd11*	At1g77300	Histone lysine 36 methyl transferase	LAZ2 is necessary for the proper expression of *LAZ5* which is then a positive mediator of *acd11*-dependent cell death	Palma et al., 2010
*laz5*	*acd11*	At5g44870	Putative TIR-NB-LRR R-protein	LAZ5 is A R-protein which could triggers cell death in the absence of ACD11	Palma et al., 2010
*lol1*	*lsd1*	At1g32540	Protein homologs to LSD1, with 3 zinc finger domains	LOL1 is and LSD1 have antagonistic effects on ROS homeostasis	Epple et al., 2003
*mc1*	*lsd1*	At1g02170	Type 1 metacaspase	Unknown	Coll et al., 2010
*oxt6*	*cat2*	At1g30460	Subunit of the CPSF complex involved in mRNA polyadenylation (CPSF30)	CPSF30 regulates positively the expression of a large number of genes involved immune responses	Bruggeman et al., 2014
	*cpr5*				
	*lsd1*				
	*mips1*				
*plp2*	*vad1*	At2g26560	Lipid acyl hydrolase with wide substrate specificities	PLP2 could provide fatty acid precursors for the biosynthesis of oxylipins	La Camera et al., 2009
*prl1*	*flu*	At4g15900	Nuclear WD40 protein with unknown molecular function	PRL1 has a role in modeling ^1^O_2_-dependent signaling	Baruah et al., 2009
*rar1*	*ssi4*	At5g51700	Small protein with two zinc binding domains, involved in defense signaling	A disruption of RAR1 affects a multiple R gene-triggered responses	Zhou et al., 2008
*rdc8*	*ssi2*	At2g40690	Glycerol-3-phosphate deshydrogenase (GLY1)	Decrease in glycerol-3-phosphate pool	Kachroo et al., 2004
*sdf2*	*bir1*	At2g25110	Protein involved in the ER quality control	Same as *crt3* suppressor	Sun et al., 2014
*sfd4*	*ssi2*	At4g30950	Fatty acid desaturase involved in the synthesis of polyunsaturated lipids	Decrease in plastid complex lipid species containing hexadecatrienoic acids	Nandi et al., 2003
*sobir1*	*bir1*	At2g31880	Receptor-like kinase	In absence of BIR1, SOBIR1 activates resistance signaling pathways	Gao et al., 2009
*soldat8*	*flu*	At2g36990	SIGMA6 (SIG6) factor of the plastid RNA polymerase	Disruption of SIG6 disturbs chloroplasts homeostasis that could acclimate plants and then suppress *flu* ^1^O_2_-mediated cell death	Coll et al., 2009
*soldat10*	*flu*	At2g03050	Plastidial protein related to the human mTERF	Same as soldat8	Meskauskiene et al., 2009
*sr1-4D (gof)*	*edr2*	At2g22300	Calmodulin-binding transcription factor	Probalby due to enhanced repression of genes involved in *edr2* cell death	Nie et al., 2012
*summ1*	*mkk1/mkk2*	At4g08480	MAP kinase kinase kinase	In absence of MKK1/MKK2 or MPK4, SUMM1 triggers SUMM2-mediated immune responses	Kong et al., 2012
	*mpk4*				
*summ2*	*mkk1/mkk2*	At1g12280	Putative NB-LRR R protein	In absence of MKK1/MKK2 or MPK4, SUMM2 activates resistance signaling pathways	Zhang et al., 2012
	*mpk4*				
*sup6*	*acd6*	At5g20660	Putative Metalloprotease	Unknown	Lu et al., 2009
*ulf3/hy1*	*flu*	At2g26670	Heme oxygenase involved in biosynthesis of chromophore	Accumulation of heme in *hy1* inhibits the Glu-tRNA enzyme conducing to a decrease in Pchlide content	Goslings et al., 2004

Together these two types of approaches have greatly contributed to our understanding of the cellular mechanisms governing PCD in plants. This review is mainly focused on the cellular functions or processes that can be grouped in several categories (i) cellular activities linked to chloroplasts activity and light energy capture controlling the onset of PCD, (ii) sphingolipids and fatty acids as regulators of cell death, (iii), signal perception at the plasma membrane that required efficient membrane trafficking (iv) secondary messengers such as ion fluxes and ROS and (v) changes in gene expression as the last integrator of the signaling pathways.

## Chloroplasts and light are hubs of stress leading to PCD

Plants need light as the source of energy for photosynthesis, but excess energy can lead to cell damage, cell death and ultimately, plant death. The severity of the symptoms of several LMM, such as *lesion simulating disease1* (*lsd1)* (Mateo et al., 2004), *myo-inositol-1-phosphate synthase1* (*mips1)* (Meng et al., 2009) and *catalase2* (*cat2)* (Queval et al., 2007), is light-dependent, suggesting that chloroplast activity can function as a signal triggering cell death, possibly via ROS production or changes in their redox state. In line with this hypothesis, several LMM mutants are deficient for chlorophyll biosynthesis or degradation, leading to abnormal accumulation of photoreactive molecules and ROS production in the light.

### Tight regulation of chlorophyll biosynthesis and degradation is essential to cell survival

**Figure 1:**
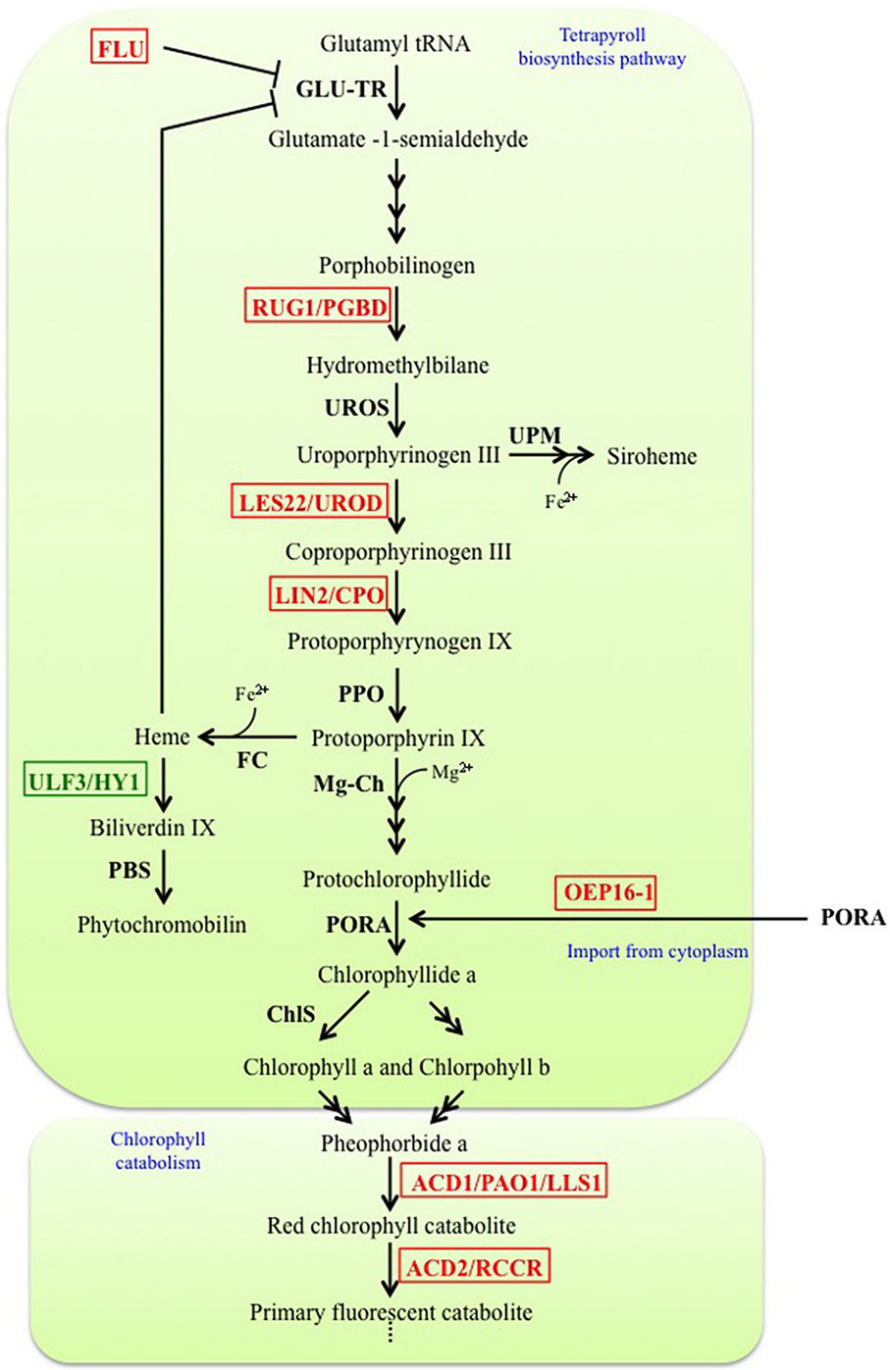
**Simplified representation of the tetrapyrrole biosynthesis pathway and chlorophyll catabolism into the chloroplast**. Factors disrupted in LMM are indicated in red whereas factor disrupted in suppressors of LMM are in green. ACD1/PAO1/LLS1, Accelerated cell death 1/Pheophorbide a oxygenase/Lethal leaf spot1; ACD2/RCCR, Accelerated cell death 2/Red chlorophyll catabolite reductase; ChlS, Chlorophyll synthase; FC, Fe chelatase; FLU, Fluorescent; GLU-TR, Glutamyl-tRNA reductase; LES22/UROD, Lesion 22/Uroporphyrinogen III decarboxylase; LIN2/CPO, Lesion initiation 2/Coproporphyrinogen III oxidase; Mg-Ch, Mg chelatase; OEP16-1, Outer plastid envelope protein 16-1; PBS, Phytochromobilin synthase; PORA, NADPH-protochlorophyllide oxidoreductase; PPO, Protoporphyrinogen IX oxidase; RUG1/PGBD, Rugosa 1/Porphobilinogen deaminase; ULF3/HY1, FLU3 written backwards/Heme oxygenase 1; UPM, Uroporphyrinogen III methylase; UROS, Uroporphyrinogen III synthase.

The tetrapyrrole biosynthesis pathway is well characterized. It takes place in chloroplasts and through branching leads to production of chlorophyll a/b, hemes, siroheme and phytochromobilin molecules (Mochizuki et al., 2010). Disruption of tetrapyrroles biosynthesis at different stages can lead to lesion-mimic phenotypes due to the abnormal accumulation of photoreactive molecules (Figure 1). For example, the mutant *rugosa1* (*rug1)* is affected in a porphibilinogen deaminase (PGBD), and accumulates porphibilinogen (Quesada et al., 2013). Likewise, accumulation of coproporphyrinogen III in *lesion initiation 2/rice lesion initiation 1 (lin2/rlin1)* mutants (Ishikawa et al., 2001; Sun et al., 2011), uroporphyrynogen III in lesion22 (*les22)* mutant (Hu et al., 1998), and protochlorophyllide (Pchlide) in *oep16* and *flu* mutants (Meskauskiene et al., 2001; Samol et al., 2011b) leads to cell death phenotypes.

The *oep16* (Samol et al., 2011b) and *flu* (Meskauskiene et al., 2001) mutants are both characterized by bleaching of seedlings when they are transferred from dark to light conditions. Unlike other LMM cited above, *outer enveloppe protein16-1* (*oep16-1)* and *fluorescent in blue light (flu)* are not mutated in genes encoding enzymes involved in the biosynthesis of tetrapyrroles, but are both characterized by over-accumulation of Pchlide in the dark. Indeed, one of the last steps of chlorophyll biosynthesis, namely the conversion of protochlorophyllide (Pchlide) to chlorophyllide (Chlide), is catalyzed by a photoenzyme and can thus not occur in the dark. Down-regulation of chlorophyll biosynthesis in the dark is therefore crucial to avoid the cytotoxic effects of the highly photo-reactive Pchlide when plants reach the light. *OEP16* encodes an amino acid-selective channel protein involved in the chloroplast import of the enzyme NADPH:Pchlide oxydoreductase A which converts Pchlide to chlorophyllide a (Samol et al., 2011b). The *FLU* gene encodes a protein with a coiled-coil TPR domain localized in the chloroplast. FLU acts negatively on the biosynthesis of tetrapyrroles in the dark by inhibiting the activity of Glutamyl-tRNA reductase (GLU-TR) (Meskauskiene and Apel, 2002; Goslings et al., 2004; Apitz et al., 2014), which catalyzes the first step of the tetrapyrrole biosynthetic pathway, thereby preventing Pchlide accumulation. Inhibition of the GLU-TR activity is also observed in the suppressor *ulf3*/*heme oxygenase 1 ulf3/hy1* because of an accumulation of heme, which then prevents Pchlide increase and suppresses cell death in the *flu* mutant (Goslings et al., 2004). Therefore, FLU appears as a key negative regulator of tetrapyrrole biosynthesis pathway in the dark, and it has been further confirmed by the interaction of FLU with several enzymes of Mg^2+^ branch of the tetrapyrroles biosynthesis pathway (Kauss et al., 2012). When *flu* and *oep16* mutants are transferred in the light after a prolonged dark period, Pchlide rapidly generates singlet oxygen (^1^O_2_) (Meskauskiene et al., 2001; Op Den Camp et al., 2003; Samol et al., 2011a). In *flu*, the accumulation of this ROS causes the loss of chloroplast integrity followed by a rupture of the central vacuole and finally cell death (Op Den Camp et al., 2003).

Surprisingly, defects in chlorophyll catabolism can also lead to cell death. Indeed, disruption of two enzymes involved in the degradation of chlorophyll generates spontaneous lesions in the *accelerated cell death1/lethal-leaf spot1 (acd1/lls1)* and *accelerated cell death2 (acd2)* LMM (Greenberg and Ausubel, 1993; Mach et al., 2001; Tanaka et al., 2003). *acd1* is characterized by an accumulation of pheophorbide (Pheide a) due to disruption of *ACD1/LLS1/PHEIDE A OXYGENASE1 (PAO1)* gene, a Pheide a oxygenase converting Pheide a into red chlorophyll catabolite (RCC) (Gray et al., 1997; Pruzinska et al., 2003; Tanaka et al., 2003). The accumulation of the photoreactive Pheide a induces cell death in an age-dependent (Pruzinska et al., 2005), and, surprisingly, also in a light-independent manner (Hirashima et al., 2009). The *ACD2/RED CHLOROPHYLL CATABOLITE REDUCTASE* (*RCCR)* gene encodes a RCC reductase, catalyzing the next reaction that converts RCC to the primary “fluorescent” chlorophyll catabolite (Mach et al., 2001). The spontaneous cell death observed in *acd2* is correlated to an accumulation of RCC and ^1^O_2_(Pruzinska et al., 2007); moreover, ACD2/RCCR may bind to porphyrin-related molecules in mitochondria to prevent PCD (Yao and Greenberg, 2006). Interestingly, during pathogen infection, ACD2 localizes dynamically between the chloroplast and mitochondria, apparently as a protective response against chloroplast-derived ACD2 substrate molecules that can target mitochondria and induce death (Pattanayak et al., 2012).

Hence, tetrapyrrole biosynthesis and destruction need to be highly controlled to avoid accumulation of photoreactive tetrapyrroles and generation of ROS, which are damaging and/or signaling molecules capable to promote cell death. Although many LMM are affected for chlorophyll biosynthesis or degradation, it remains unclear whether changes in the accumulation of photoreactive chlorophyll precursors or degradation products actually contribute to PCD control during plant development or in response to stress. However, one major finding associated with the description of this class of LMM is the identification of PCD regulatory pathways that respond to ROS production in the chloroplast. Indeed, the triple mutant *executer1 executer2 flu* (*ex1 ex2 flu)* still accumulates Pchlide and ^1^O_2_, whereas the cell death phenotype is suppressed (Wagner et al., 2004; Lee et al., 2007b). Importantly, EX proteins have been shown to operate in the wild-type under high light stress (Kim et al., 2012). This demonstrates that PCD observed in the *flu* mutant is not a consequence of cellular damage caused by ^1^O_2_ accumulation, and that the plastidial proteins EX1 and EX2 are involved in the retrograde control of nuclear ^1^O_2_-responsive genes and are both necessary to integrate the ^1^O_2_ signal from chloroplasts to nucleus.

### Alteration of chloroplast integrity or activity can modulate PCD

Alteration of chloroplast function or integrity can lead to two opposite effects: promotion or suppression of cell death. For example, disruption of *LESION INITIATION1* (*LEN1)* and *LRGB* which encode respectively a chloroplastic chaperonin 60 β (Ishikawa et al., 2003) and an intermembrane protein localized in chloroplast envelope (Yamaguchi et al., 2012; Yang et al., 2012), leads to spontaneous cell death, but the underlying mechanisms are still unknown. The FZO-like (FZL) protein, a membrane remodeling GTPase, has a crucial role in thylakoid organization (Gao et al., 2006), and its disruption induces alteration in chloroplast number, size and shape and also the development of spontaneous cell death in *fzl* mutants, suggesting that damages to the chloroplast membranes could trigger ROS accumulation and induce cell death (Landoni et al., 2013).

Thorough analysis of the *lsd1* mutant brought further evidence for the role of chloroplast function in the control of PCD. Indeed this mutant has a conditional phenotype, and displays spontaneous lesions only in long-day condition (Jabs et al., 1996) or high illumination (Mateo et al., 2004). Lesions formation in *lsd1* has been shown to depend on the redox status of the plastoquinone pool: this mutant is incapable to confine cell death induced by over-reduction of plastoquinones due to excess excitation energy (Mateo et al., 2004; Muhlenbock et al., 2008). Consistently, a mutation in the *CHLOROPHYLL A/B BINDING HARVESTING-ORGANELLE SPECIFIC (CAO)* gene encoding a chaperone required for the assembly photosystem II antenna leads to a reduction of lesion formation in *lsd1* (Mateo et al., 2004). Likewise, inhibition of chlorophyll biosynthesis abolished cell death in another LMM: the *mips1* mutant (Meng et al., 2009) and several mutations affecting chlorophyll metabolism have been reported to suppress lesion formation in *len1* (Ishikawa, 2005). Together, these studies support the notion that perturbation of the photosynthetic metabolism can trigger PCD.

Interestingly, alteration of other chloroplast functions can also suppress cell death in some LMM. Indeed, the two suppressors of *flu*, *soldat8* and *soldat10* are mutated in genes involved in plastid RNA transcription (Coll et al., 2009; Meskauskiene et al., 2009). Both mutants display disturbance of chloroplast homeostasis that could acclimate plants to stress, thereby preventing *flu* mediated cell death. The last example of the bipolar function of chloroplasts in PCD is given by the mutant *aberrant growth and death2 (agd2)*, which is deficient for a plastidial aminotransferase (Rate and Greenberg, 2001; Song et al., 2004). Surprisingly, this LMM is suppressed by disruption of AGD2-LIKE DEFENSE RESPONSE PROTEIN 1 (ALD1), another plastidial aminotransferase (Song et al., 2004). The authors suppose that AGD2 synthesizes an amino acid–derived molecule that suppresses defenses, whereas ALD1 generates a related amino acid–derived molecule important for activating defense signaling.

Therefore, chloroplasts are the source of opposite signals able to prevent or to promote cell death, and complex retrograde signaling pathways from plastids to the nucleus are necessary to integrate them and induce appropriate cellular responses (Galvez-Valdivieso and Mullineaux, 2010; Karpinski et al., 2013).

Hence, forward genetic approaches have identified chloroplasts as likely sources of pro-death signals. Indeed, because metabolic pathways occurring in chloroplasts and in particular photosynthesis are exquisitely sensitive to changes in environmental conditions, these organelles appear ideally suited to play a prominent role in stress perception. However, stress perception can occur in various other cellular compartments, notably in cellular membranes which are exposed at early steps of pathogen infections.

## Several classes of lipids are key molecules controlling plant PCD

Sphingolipids and oxylipins both possess Fatty Acids (FA) in the composition of their skeleton. Because of their chemical diversity they have the potential to fine-tune many cellular processes. In this section, we will address how the characterization of some specific LMM highlighted the fundamental role of these molecules in the control of plants PCD.

### Sphingolipids as inducers of PCD

**Figure 2:**
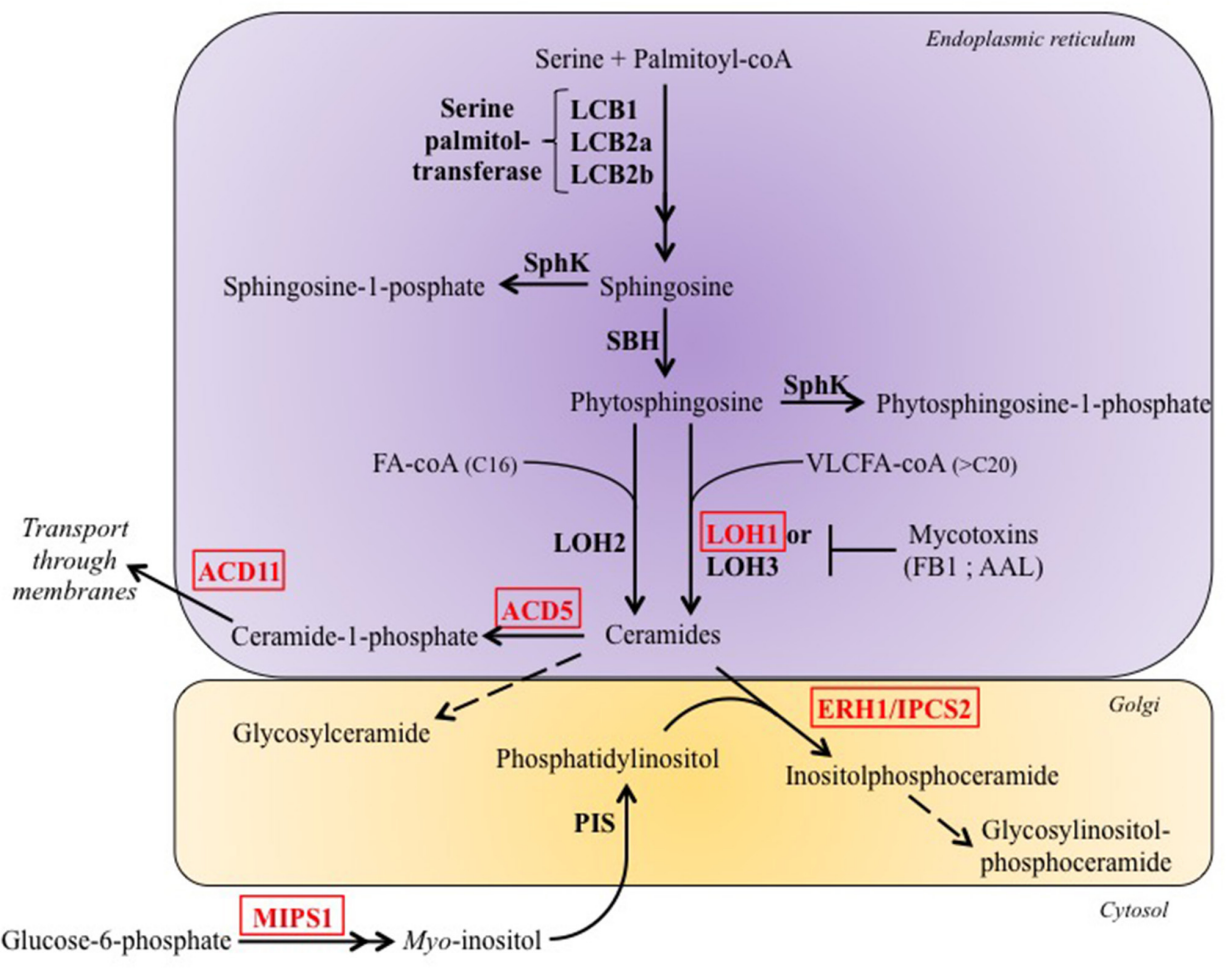
**Simplified representation of sphingolipid metabolism in plants**. The *de novo* biosynthesis of ceramides occurs in the endoplasmic reticulum and synthesis of more complex sphingolipids occurs in the Golgi. Sphingosine and phytosphingosine are both referred in the text as Long-chain Basis (LCB). Factors disrupted in LMM are indicated in red. AAL, Alternaria alternata f. sp. lycopersici; ACD5/11, Accelerated cell death 5/11, are a ceramide kinase and a ceramide-1-phosphate transporter, respectively; ERH1/IPCS2, Enhancing RPW8-mediated HR-like cell death 1/Inositolphosphoceramide synthase 2; FB1, Fumosin B1; LCB1/2a/2b, Long-chain base 1/2a/2b, are subunits which form the Serine palmitol-transferase; LOH1/2/2 Lag one homolog1/2/3, are ceramide synthases; MIPS1, Myo-inositol-1-phosphate synthase; PIS, Phosphatidylinositol synthase; SBH, Sphingoid base hydroxylase; SphK, Sphingosin kinase.

Sphingolipids are a class of lipids present ubiquitously in a large variety of organisms including eukaryotes and bacteria. All complex sphingolipids are composed of a polar head group and an *N*-acetyl FA linked to a sphingoid long chain base (LCB), via the amine bond, to form the basic unit of all sphingolipids: the ceramide (Pata et al., 2010). Sphingolipids display a high structural diversity caused by variability in the length of the sphingoid LCB and the *N*-acetyl FA, in the number of saturations, in the degree of hydroxylation, and in the diversity of the head group (Pata et al., 2010). Their biosynthesis starts in the endoplasmic reticulum (ER) and ends in the Golgi apparatus (Figure 2).

Several LMM are characterized by over-accumulation of ceramides due to the disruption of enzymes that convert them in other molecules. It is the case of the *accelerated cell death 5* (*acd5*) mutant (Greenberg et al., 2000), which is deficient for a ceramide kinase (Liang et al., 2003) and of the *enhancing RPW8-mediated HR-lihe cell death 1* (*erh1*) which lacks the inositol-phosphorylceramide synthase 2 (IPCS2). Quantitative sphingolipid profiling indicated that ceramide accumulation in *acd5* paralleled the appearance of spontaneous cell death, and it was accompanied by autophagy and mitochondrial ROS accumulation (Bi et al., 2014). IPCS2 catalyzes the production of inositolphosphorylceramide (IPC) and diacylglycerol from ceramide and phosphatidylinositol (Wang et al., 2008). In the absence of this enzyme, synthesis of RESITANCE TO POWDERY MILDEW 8 (RPW8), a protein that confers resistance to powdery mildew, induces an accumulation of ceramides and massive HR-like cell death (Wang et al., 2008). Moreover, the *acd5 erh1* double mutant showed an additive effect with more severe cell death (Wang et al., 2008), likely as a consequence of higher levels of ceramides.

The relative accumulation of ceramides and their derivatives thus appear as key elements for the control of plant PCD. Indeed, ceramide-phosphate partially blocks cell death induced by ceramides (Liang et al., 2003), suggesting that ceramides are inducers of PCD whereas their phosphorylated forms block it. This is further supported by the observation that Arabidopsis mutant of the *MYO-INOSITOL-1-PHOSPHATE SYNTHASE 1* (*MIPS1*) forms spontaneous lesions (Meng et al., 2009; Donahue et al., 2010; Luo et al., 2011). Indeed, *myo*-inositol is used to synthesize numerous compounds in the cell, including phosphatidylinositol, and inhibition of *myo*-inositol production could thus mimic *erh1* PCD (Wang et al., 2008) by preventing the conversion of ceramides in IPC. In agreement with this hypothesis, elevated levels of ceramides and hydroxyceramides were observed in *mips1* (Donahue et al., 2010).

Interestingly, membrane transfer of sphingolipid also seems to play a crucial role in regulating plant PCD. Indeed, it has been recently shown that *acd11*, which displays chlorosis that can lead to plant death (Brodersen et al., 2002), is mutated in a ceramide-1-phosphate (C1P) transfer protein (Simanshu et al., 2014). ACD11 selectively transfers C1P between membranes, and disruption of this activity in *acd11* leads to constitutive accumulation of C1P but an even stronger elevation of non-phosphorylated ceramides is observed (Simanshu et al., 2014), which may be responsible for the induction of cell death.

It is worth noting that the phosphatidylinositol derivatives phosphoinositides are known to be involved in the transport of sphingolipids. For example, the mammalian ceramide transport protein CERT requires direct binding to phosphatidylinositol-4-phosphate for its function (Hanada et al., 2003). Thus, defects in phosphatidylinositol-4-phosphate accumulation in *mips1* may impact ceramide transport as well as their metabolism. Nevertheless, because *myo*-inositol is involved in a large number and diverse cellular processes (Valluru and Van Den Ende, 2011), it is possible that spontaneous cell death observed in *mips1* is not directly the consequence of sphingolipids accumulation.

An additional layer of complexity comes from the fact that disruption of *LAG ONE HOMOLOG 1* (*LOH1*) gene, encoding the ceramide synthase involved in the production of very long chain fatty acids (VLCFA) containing ceramides conduces to spontaneous cell death (Ternes et al., 2011). This finding is consistent with the early observation that the bacterial toxins secreted by necrotrophic pathogens: AAL (named from initials of the producing pathogen, *Alternaria alternata* f. sp. *lycopersici*) and Fumonisin B1 (FB1) (Merrill et al., 1993; Wang et al., 1996) target LOHs, and suggests that different families of ceramides may play distinct roles in the control of PCD. Indeed, AAL and FB1 do not target LOH2 which is involved in the synthesis of ceramides containing C16 fatty acids (Markham et al., 2011) and *loh1* is characterized by increased levels of LCB, as well as of C16 containing ceramides, but not of VLCFA containing ceramides, indicating that in this case, spontaneous cell death is triggered either by the accumulation of LCB or C16 containing ceramides (Ternes et al., 2011).

Taken together, these studies on LMM indicate that LCB and ceramides are sphingolipids promoting PCD, not only in Arabidopsis but also in monocots (Bi et al., 2011). However, the underlying mechanisms are not well understood. It has been shown that pathogen-specific sphingolipids induced Ca^2+^ signaling, Mitogen Associated Protein Kinases (MAPK) activation and ROS production in cultured rice cells (Kurusu et al., 2011). Moreover, the suppressor of *acd11, lazarus5* (*laz5*) is deficient for a putative Toll-Interleukin-1 Receptor-Nucleotide Binding Site-Leucine Rich Repeat factor (TIR-NBS-LRR) (Palma et al., 2010). The TIR-NBS-LRRs are generally known to be involved in the recognition of avirulence factors (Rafiqi et al., 2009). Another link between sphingolipids and activation of cell death, is that the MPK6 protein, a positive regulator of plant defenses, has been identified as a potential target of LCBs (Saucedo-Garcia et al., 2011).

### Fatty acids and oxylipins

There is major evidence that simple FA can also regulate plant-pathogens interactions and cell death (Walley et al., 2013). The LMM *suppressor of SA insensitivity2/fatty acid biosynthesis2* (*ssi2/fab2*) is mutated in a gene encoding one of the steroyl-ACP desaturase, involved in fatty acid desaturation (Kachroo et al., 2001). In *ssi2/fab2*, a decrease in 18:1 FA leads to a stabilization of NITRIC OXIDE ASSOCIATED1 (NOA1), an enzyme that regulates NO levels, and thus to an increase in endogenous NO levels (Mandal et al., 2012). This triggers transcriptional up-regulation of NO responsive nuclear genes, thereby activating disease resistance and cell death. A mutation in *ACT1*, encoding a glycerol-3-phosphate acyltransferase, conduces to accumulation of 18:1 FA and suppresses lesions in the *ssi2 act1* double mutant, confirming the role of 18:1 in the regulation of cell death (Kachroo et al., 2003).

One of the key processes in early plant defense signaling is enhanced lipid peroxidation and production of a vast array of FA-derived compounds called oxylipins. After stimuli which activate lipases, FA such as linoleic acid are released from the chloroplastic membrane (Walley et al., 2013). Oxylipins are then produced through parallel and competing branches of the allene oxide synthase (AOS) and hydroperoxide lyase (HPL) pathways (Creelman and Mullet, 1997; Matsui, 2006). The AOS pathway is responsible for stress-inducible production of jasmonates such as jasmonic acid (JA) and methyl jasmonate (MeJA) (Creelman and Mullet, 1997). The HPL pathway produces C6-aldehydes and corresponding derivatives (Matsui, 2006). These two competing pathways are both important in the elicitation of plant defense responses against biotic agents (Walley et al., 2013): impairment of the HPL pathway in the rice LMM *constitutive expression of allene oxide synthase gene62 (cea62)* causes lesions due to overproduction of JA (Liu et al., 2012). Moreover, the PATATIN-LIKE PROTEIN 2 (PLP2), a pathogen-induced patatin-like lipid acyl hydrolase with a wide range of substrates, seems to be necessary to provide FA precursors for the biosynthesis of oxylipins which are then necessary to spontaneous vascular necrotic lesions observed in the LMM *vascular associated death 1* (*vad1*) (Lorrain et al., 2004; La Camera et al., 2009).

Taken together, these studies demonstrate that FA and oxylipins are other molecules involved in the control of cell death in plants, adding an additional layer of complexity to the control of the plant PCD by cellular metabolites.

## From immunity response and cellular trafficking to cell death

### Immunity component

In addition of PAMP-triggered immunity, another type of innate immune responses in plants is effector-triggered immunity (ETI), which evolved in plants to detect pathogen effectors and initiate defense response. ETI is mediated by the host resistance (R) genes and recognition of pathogen effectors by R proteins can be either direct or indirect (Chen et al., 2010). In Arabidopsis, R proteins are encoded by approximately 150 genes and are classified according to the domains they are built of, and a number of LMM are deficient for such proteins. The *suppressor of SA insensitivity4* (*ssi4*) LMM is a gain of function (gof) mutant affected in a TIR-NBS-LRR factor (Shirano et al., 2002). Constitutive activation of the SSI4 protein activates SA signaling pathways and induces the formation of chlorotic lesions (Zhou et al., 2004). As previously mentioned, *laz5*, a suppressor of *acd11*, is mutated in a gene encoding a putative TIR-NBS-LRR which is required to trigger cell death (Palma et al., 2010). Three other R proteins, ACTIVATED DISEASE RESISTANCE 1 (ADR1), ADR1-LIKE 1 (ADR1-L1) and ADR1-LIKE 2 (ADR1-L2), belonging to the Coiled-Coil-NBS-LRR group are also activators of defense responses and cell death via regulation of SA signaling (Bonardi et al., 2011; Roberts et al., 2013): loss of these three factors allows the suppression of the *lsd1* runaway cell death.

Analysis of LMM also allowed the identification of signaling components activated downstream of receptors involved in PTI or ETI. Upon recognition of bacterial flagellin, the plant receptor FLAGELLIN SENSING 2 (FLS2) heterodimerizes with BRASSINOSTEROID INSENSITIVE 1-ASSOCIATED RECEPTOR KINASE 1 (BAK1) and activates plant defense responses via two parallel MAPK cascades, one involving MPK3 and MPK6 that positively regulate defense responses and one involving MPK4, a negative regulator of R protein signaling (Colcombet and Hirt, 2008; Rasmussen et al., 2012). Indeed, both the *mpk4* and the *mkk1/mkk2* mutant, which is deficient for the upstream MAPKKs, are LMM that show drastically reduced growth and spontaneous cell death on leaves. These two LMM are suppressed by mutations affecting a MAP kinase kinase kinase (*suppressor of mkk1 mkk2 1*) and a putative NB-LRR R protein (*summ 2*) (Kong et al., 2012; Zhang et al., 2012). The authors assumed that the absence of the MKK1/MKK2 or MPK4 protein activates SUMM1, which then triggers SUMM2-mediated immune responses.

Disruption of BIR1, a BAK1-interacting receptor-like kinase, leads to extensive cell death, activation of constitutive defense responses and impairment in the activation of MPK4 (Gao et al., 2009). It likely functions by antagonizing the activity of another RLK: SUPPRESSOR OF BIR1 (SOBIR1). Indeed, disruption of *SOBIR1* suppresses lesion formation in *bir1* whereas its over-expression in a wild-type context triggers cell death and immune responses signaling pathways (Gao et al., 2009).

Others examples highlight the crucial role of R proteins in regulating cell death in plants. Indeed, constitutive activation of the protein RECOGNITION OF *PERONOSPORA PARASITICA* 4 (RPP4), a TIR-NBS-LRR, in the *chilling-sensitive 2* mutant (*chs2*), leads to the development of lesions in low temperature condition (Huang et al., 2010). On the contrary, the disruption of a TIR-NBS-LRR protein with a WRKY domain, in the mutant *sensitive to low humidity 1* (*slh1*) induces the apparition of spontaneous lesions (Noutoshi et al., 2005).

One important mechanism controlling the activity of these membrane bound receptors appears to be vesicular trafficking. Indeed, it has been demonstrated that systemic acquired resistance (SAR) requires the regulation of the secretory pathway by the NONEXPRESSER of PR1 (NPR1) protein that directly binds the promoters of genes involved in this pathway (Wang et al., 2005).

### Intracellular trafficking

Additional evidence for an involvement of subcellular trafficking in the immune responses and cell death came from the isolation of the *exocyst subunit EXO70 family protein B1 (exo70b1)*, *enhanced disease resistance3* (*edr3*) and *spotted lesion28* (*spl28*) LMM mutants which are all impaired in vesicular trafficking. The *EX70B1* gene encodes a subunit of the exocyst complex (Kulich et al., 2013; Stegmann et al., 2013), whereas *edr3* is mutated in the dynamin related protein 1E (DRP1E) which could serve to attach vesicles to microtubules (Hong et al., 2003) and *SPL28* encodes a clathrin adaptator subunit which may be involved in vesicular trafficking in the post-golgi (Qiao et al., 2010). However, the mechanisms connecting vesicular traffic to the observed spontaneous lesions remain to be elucidated. EXO70B1 may have a positive role for internalization of autophagic bodies in the vacuole (Kulich et al., 2013). Autophagy is a well-documented process with crucial roles in development, immune responses and PCD. For thorough information of this subject, the reader is referred to other reviews (Patel et al., 2006; Yoshimoto, 2010; Hofius et al., 2011). Nevertheless, the mutant *exo70b1* is the only autophagic mutant with a known lesion-mimic phenotype, suggesting that mechanisms underlying lesion formation in other mutants deficient for vesicular trafficking may be unrelated to autophagy.

Interestingly, some LMM suppressors are also involved in intracellular trafficking: *calreticulin3* (*crt3*), *ER-localized dnaJ-like protein 3b* (*erdj3b*) and *stromal-derived factor 2* (*sdf2*) that have been described as suppressors of *bir1* are all mutated in genes involved in the ER quality control (Sun et al., 2014). Disruption of *CRT3*, *ERDJ3B* or *SDF2* conduces to a reduced accumulation of SOBIR1 (Sun et al., 2014), indicating that the secretory pathway of ER quality control plays important roles in the biogenesis of SOBIR1 and is required for cell death in *bir1*. Moreover, the *acd6* LMM (Rate et al., 1999) harbors a gof mutation in a gene encoding a membrane protein with an ankyrin domain (Lu et al., 2003). ACD6 is localized in the ER and the plasma membrane where it forms complexes with FLS2 and BAK1 (Zhang et al., 2014b). SA signaling increases the plasma membrane pools of ACD6, FLS2, and BAK1, thus, authors suppose that SA improves the efficiency of productive folding and/or complex formation in the ER, such that ACD6, FLS2, and BAK1 reach the cell surface to more effectively promote immune responses (Zhang et al., 2014b). Therefore, ER quality control is an emerging regulatory process of plant defenses via regulation of the abundance and quality of transmembrane receptors and modulation of signal downstream of the receptors (Tintor and Saijo, 2014).

Simultaneous disruption of SYNTAXIN OF PLANTS 121 and 122 (SYP121 and 122), members of the SNARE machinery, in the *syp121/syp122* double mutant, leads to constitutive activation of defense responses (Zhang et al., 2008). This activation is dependent of numerous R proteins and results in a dwarf phenotype and the development of lesions.

Together, these studies demonstrate that polarized trafficking and secretory pathways are crucial in the immune and cell death processes. This is supported by the fact that one of the first subcellular response of plants upon detection of pathogens is the reorganization and polarization of cytoskeleton to the site of attack (Hardham et al., 2007), and that RPW8.2, is specifically targeted to the extrashaustrial membrane, the host-pathogen interface (Wang et al., 2009).

## Ion fluxes and reactive oxygen species contribute to signal transduction leading to cell death

As seen above, stress can be perceived by plants in a variety of ways depending on its nature. Then, mechanisms such as ionic flux, ROS production and kinase signaling pathways conduce to activation of plant defenses (Muthamilarasan and Prasad, 2013). This part describes how LMMs proved to be powerful tools to decipher all these defense mechanisms.

### Signal transduction via ion fluxes

One of the first responses after pathogen recognition is the modification of ionic fluxes. In particular, a high influx of the calcium ion (Ca^2+^) in the cytoplasm plays a pivotal role in mediating plant immune processes, including stomatal closure, SA production and control of ROS accumulation (Ogasawara et al., 2008; Bashir et al., 2013; Baxter et al., 2014). This influx is then detected by sensors called calmodulins, that directly regulate the activity of target proteins.

Some LMM, such as *constitutive expressor of PR genes22* (*cpr22*), *death no death1* (*dnd1*) or *dnd2*/ *HR-like lesion mimic1* (*hlm1*) are mutated in genes encoding ion channels allowing the influx or efflux of ions. In *cpr22*, a deletion of 3 kb between two adjacent genes, named *CYCLIC NUCLEOTIDE-GATED ION CHANNEL 11* and *12* (*CNGC11* and *12*), fuses them and creates a chimeric and constitutively active protein CNGC11/12 responsible for lesions (Yoshioka et al., 2001, 2006). CNGC11 and 12 may act as ion channels for Ca^2+^, and the phenotype of *cpr22* might be due to activation of Ca^2+^ dependent signaling because channel blockers for this cation inhibit lesions formation (Urquhart et al., 2007). Interestingly, several suppressors of *cpr22* are in fact mutated in the chimeric protein CNGC11/12, underlying the importance of some residues for the functionality of the ion channel (Baxter et al., 2008; Chin et al., 2010; Abdel-Hamid et al., 2013). Surprisingly, in *dnd1*, disruption of *CNGC2*, encoding another calcic channel (Yu et al., 1998; Clough et al., 2000; Ali et al., 2007) causes opposite phenotypes, namely constitutive activation of broad-spectrum defenses and rare necrotic lesions on the one hand, and disruption of HR cell death on the other hand (Yu et al., 1998; Ahn, 2007). To account for this apparent contradiction, Clough et al. (2000) have postulated that unbalanced ionic homeostasis in *dnd1* could mimic a pathogen attack and induce defense response genes, among which catalase and ascorbate peroxidase, that could in turn block HR-related cellular processes (Clough et al., 2000). Furthermore, the lack of HR in *dnd1* could be explained by the fact that a Ca^2+^ influx generated by CNGC2 is necessary to the accumulation of NO and subsequent lesion formation (Ali et al., 2007). CNGC2 physically interacts with another ion channel, CNGC4/HLM1, and they act together to regulate immune responses and floral transition (Chin et al., 2013). Disruption of *CNGC4/HLM1*, in the *dnd2/hlm1* mutant, causes similar phenotypes to *dnd1* (Balague et al., 2003; Jurkowski et al., 2004), but unlike CNGC2 the HLM1/CNGC4 protein, which has one ortholog identified in barley (Rostoks et al., 2006; Keisa et al., 2011), has specificity for potassium (K^+^) and sodium (Na^+^) (Balague et al., 2003).

LMMs also allowed the identification of factors acting downstream of ion signaling. The *COPINE/BONZAI* (*CPN/BON*) gene family encodes Ca^2+^-dependent, phospholipid-binding proteins that repress cell death (Yang et al., 2006). *cpn1*/*bon1* mutant displays lesions due to the over-accumulation of the TIR-NBS-LRR protein SUPPRESSOR OF NPR1-1 CONSTITUTIVE 1 (SNC1) (Jambunathan et al., 2001; Yang and Hua, 2004). Thanks to its C2 and Willerbrand A domains, involved in Ca^2+^-dependent phospholipid binding and protein binding, respectively, CPN1/BON1 is localized at plasma membrane (Li et al., 2010) and could recruit partners to the membrane to suppress SNC1 activation (Liu et al., 2005). In addition, *cpn1/bon1* is suppressed by mutations in putative R genes (Li et al., 2009), and CPN1/BON1 interacts with the RLKs BAK1 and BIR1 and is phosphorylated by BAK1 (Wang et al., 2011). Given these findings, CPN1/BON1 is probably a signaling protein that integrates calcium and RLKs in the signal transduction pathways involved in cell death and defense responses.

### ROS signaling

As mentioned above, ROS production caused by adverse environmental conditions likely functions to trigger PCD. In addition, ROS play a complex role as secondary messengers in the signaling pathways leading to PCD. Their role has been studied in detail in the *lsd1* mutant, and these studies led to the conclusion that ROS can have opposing effects on PCD depending on the cellular compartment in which they are produced. *LSD1* encodes a protein with three zinc finger domains (Dietrich et al., 1997) and can interact with the three Arabidopsis catalases (Li et al., 2013) that are responsible for the detoxification of the peroxysomal H_2_O_2_. These interactions are necessary for a proper catalase activity, and disruption of *LSD1* conduces to a decrease in catalase activity (Mateo et al., 2004; Li et al., 2013) thereby triggering cell death. This mechanism is further supported by the observation that mutants deficient for the main leaf catalase CAT2 show high increase in H_2_O_2_ cellular content and the apparition of lesions only in long-day conditions (Vandenabeele et al., 2004; Queval et al., 2007). Consistently, inhibition of photorespiration under high CO_2_ or low O_2_ conditions decreases the number and the intensity of lesions in *lsd1* (Mateo et al., 2004).

H_2_O_2_ production in the peroxisome thus appears to trigger PCD. By contrast, apoplastic ROS production may inhibit PCD. In the apoplast, superoxide is produced by transmembrane NADPH oxydases using dioxygen as substrate, and then it is converted in H_2_O_2_ by Superoxide dismutases (SOD) (Apel and Hirt, 2004). The *lsd1* mutant is affected in the induction of *SOD* genes which may conduce to high accumulation of superoxide and the runaway cell death (Kliebenstein et al., 1999). However, contrary to what expected, disruption of the two NADPH oxydases, RESPIRATORY BURST OXIDASE HOMOLOG D and F (RbohD and F), does not suppress lesions in *lsd1*. Indeed, cell death is enhanced in the double mutants *lsd1 rbohd* and *lsd1 rbohf* and the triple mutant *lsd1 rbohd rbohf* is lethal (Torres et al., 2005). Likewise, disruption of the *RBOHf* gene aggravates lesion formation in the *cat2 rbohf* double mutant (Chaouch et al., 2012). RbohD and F thus appear as negative regulators of cell death propagation although they are likely to be positive regulators of PCD initiation (Chaouch et al., 2012). How ROS production connects with downstream mechanisms leading to cell death remains to be fully elucidated. Interestingly, a decrease in *myo*-inositol accumulation is necessary for the development of lesions in the *cat2* mutant (Chaouch and Noctor, 2010), providing evidence for a potential link between ROS- and ceramide-dependent signaling.

Therefore, Ca^2+^ and ROS are key signal molecules for the initiation and the spreading of plant PCD. The LSD1 protein is a crucial hub regulating at once extracellular and intracellular ROS production that allows plants to integrate and coordinate the perception of different biotic and abiotic stresses via profound changes in the expression of the genome.

## Integrating signals in the nucleus to modulate gene expression

PCD is an active process that requires changes in nuclear gene expression. Isolation of various LMM or suppressors of LMM has shed light on the role of transcription factors, chromatin modifications, and post-transcriptional factors in the control of PCD.

### Modulating gene transcription

The CALMODULIN-BINDING TRANSCRIPTION FACTOR 3/sIGNAL RESPONSIVE 1 (CAMTA3/SR1) transcription factor can integrate several PCD signaling pathways. Its tobacco ortholog, ETHYLENe-UP-REGULATED 1 (ER1), was firstly characterized as a protein involved in plant senescence controlled by ETH (Yang and Poovaiah, 2000), and the *Arabidopsis* CAMTA3/SR1 was subsequently shown to interact with calmodulin in a Ca^2+^ dependent manner and to bind DNA CGCG *cis*-elements (Yang and Poovaiah, 2002). The involvement of CAMTA3/SR1 in the regulation of plant cell death came later with the characterization of *camta3/sr1* mutants which display spontaneous chlorotic lesions on rosette leaves (Galon et al., 2008). Further studies demonstrated that CAMTA3/SR1 directly represses the expression of *ENHANCED DISEASE SUSCEPTIBILITY 1* (*EDS1*) and *ETHYLENE-INSENSITIVE 3* (*EIN3*), encoding two crucial positive regulators of SA and ETH signaling pathways (Du et al., 2009; Nie et al., 2012). Moreover, during pathogen attack, CAMTA3/SR1 interacts with the SR1 INTERACTION PROTEIN 1 (SR1IP1), a ubiquitin E3 ligase, which mediates its degradation, thus removing the repression on *EDS1* (Zhang et al., 2014a). Therefore, CAMTA3/SR1 is a Ca^2+^/calmodulin-dependent transcription factor with an inhibitory function in plant defense and cell death (Du et al., 2009).

Although the LSD1 protein displays three zinc finger domains potentially involved in DNA binding, there is no clear evidence for its role as a transcription factor. Nevertheless, LSD1 could act indirectly on gene expression by modulating the activity of other transcription factors, such as the basic leucine zipper 10 transcription factor (bZIP10). bZIP10 binds on C- and G-box DNA sequences and its disruption partially suppresses the *lsd1* phenotype (Kaminaka et al., 2006). LSD1 interacts with bZIP10 and retains it outside of the nucleus. After perception of an appropriate reactive oxygen-derived signal, or in the *lsd1* mutant, bZIP10 is dissociated from LSD1 and translocates into the nucleus (Kaminaka et al., 2006), where it may induce the expression of HR- and defense-related genes. Moreover, the *LSD1-LIKE 1* (*LOL1*) gene, acts antagonistically to LSD1 to promote cell death, and its mutation suppresses *lsd1* runaway cell death (Epple et al., 2003). These antagonism mechanisms between LSD1, bZIP10, and LOL1, enable fine-tuning of immune responses and cell death mediated by ROS.

The rice LMM *lesion mimic and senescence* (*lms)*, displaying reddish-brown lesions on leaves and rapid senescence after flowering, is also mutated in a transcriptional regulator (Undan et al., 2012). *LMS* encodes a protein with a CTD phosphatase domain involved in the dephosphorylation of the CTD of the largest subunit of the RNA polymerase II, thereby regulating transcription of target genes.

The *caa39* mutant, a suppressor of *flu* (Simkova et al., 2012), corresponds to a weak mutant allele of the *TOPOISOMERASE VI A-SUBUNIT* (*TOP6A*). TOP6A binds to the promoters of ^1^O_2_-responsive genes, and hence could directly regulate their expression (Simkova et al., 2012). TOP6A could therefore act as an integrator of signals generated by ROS formed under adverse environmental conditions.

There is also accumulating evidence for the role of chromatin modifications in the control of plant immunity (Alvarez et al., 2010), some of which come from the characterization of LMM. Mutation in the histone methyl transferase *LAZARUS 2/SET DOMAIN GROUP 8* (*LAZ2/SDG8*), suppresses the cell death occurring in *acd11* (Palma et al., 2010). The LAZ2/SDG8 protein binds to the *LAZ5* gene, encoding a putative TIR-NBS-LRR protein, and methylates histone H3 on lysine 36. This is necessary for the proper expression of *LAZ5*, the development of cell death in *acd11*, and for basal and R protein-mediated resistance in *Arabidopsis* (Palma et al., 2010). Moreover, MIPS1 has a dual function: it is involved in primary metabolism via synthesis of in *myo*-inositol but can also be imported into the nucleus where it regulates its own expression (Latrasse et al., 2013). Indeed, MIPS1 binds directly to its own promoter to stimulate transcription by locally inhibiting the spreading of Arabidopsis THRITORAX RELATED 5 and 6 (ATXR5 and 6)-dependent heterochromatin marks coming from a transposable element. Furthermore, upon activation of pathogen response, the inhibitory action of *MIPS1* on ATXR5/6 is alleviated and expression of *MIPS1* decreases (Latrasse et al., 2013).

### Post-transcriptional factors

The final amount of proteins can also be regulated by mRNA processing and export and several suppressors of LMM are affected in these processes.

The *pleitropic locus1* (*prl1)* mutant, a suppressor of *flu*, is mutated in a gene encoding a protein with a putative function in pre-mRNA splicing (Baruah et al., 2009). Therefore, the PRL1 protein may control the expressions of ^1^O_2_-responsive genes to promote cell death occurring in *flu*.

Recently, the polyadenylation factor subunit Cleavage and polyadenylation specificity factor 30 (CPSF30) has been proposed as a key component of plant PCD (Bruggeman et al., 2014). Indeed, the *oxidative stress tolerance6* (*oxt6*) mutant, with a T-DNA disrupting the gene *CPSF30*, initially identified by the authors as a suppressor of *mips1* also fully suppresses the phenotype of four other LMM: *constitutive expressor of PR genes5* (*cpr5*), *cat2*, *lsd1* and *mpk4*. Transcriptomic analyses showed that CPSF30, while it is involved in a general process, is specifically required for the proper expression of a large number of genes involved in mechanisms such as defense responses, cell death and abiotic stress responses (Bruggeman et al., 2014) and appears to be required for a correct polyadenylation site choice of the pre-mRNA transcripted from these genes (Thomas et al., 2012; Bruggeman et al., 2014).

Nonsense-mediated RNA decay (NMD) is an important surveillance mechanism that eliminates transcripts with nonsense mutations (Isken and Maquat, 2008). The UPF1 and SMG7 proteins are core components involved in NMD. The analysis of several mutants deficient for these two proteins shows that impaired NMD elicits defense responses and LMM phenotypes which appear to correlate with the extent of NMD deficiency (Riehs-Kearnan et al., 2012).

The LMM *enhanced disease resistance1* (*edr1*) which displays spontaneous cell death in the presence of the biotrophic powdery mildew pathogen, is suppressed by a mutation of the gene *HEPARANASE 1* (*HPR1*), a homolog of human *HPR1*, which is a component of the THO/TREX complex (Pan et al., 2012). The THO/TREX complex functions in mRNA processing and export. Confirming this, the mRNA export is compromised in the Arabidopsis *hpr1* mutant (Pan et al., 2012).

All together, these studies on suppressors of LMMs, and others studies (Gong et al., 2005; Germain et al., 2010; Lee et al., 2012; Vi et al., 2013), clearly highlight the requirement of a functional mRNA processing and export machinery to induce cell death and proper defense responses. Although such a mechanism has never been described to date, it is tempting to speculate that plant pathogens could secret effectors to inhibit this processes, as shown for the human CPSF30 that is targeted by the Nonstructural Protein1 (NS1) of the *Influenza A* virus, leading to a blockage of the host innate immune response (Das et al., 2008).

## Conclusions and perspectives

Genetic approaches through the identification and characterization of LMMs and their related suppressors have enabled to better understand several intra- and extracellular mechanisms involved in fine-tuning plant PCD. Whereas several of these mutants have putative roles in ceramide signaling or chloroplastic function, R proteins are a significant type associated with LMM. Knockout mutants that eliminate host guards mimic the effects of pathogen effectors, and have been found to exhibit *R*-gene-dependent cell death. Therefore, it is possible that many LMM may correspond to gene functions that are guarded by R-proteins. If so, the diverse functions of these genes may be not directly related to PCD but only implicated in this process due to their targeting by pathogen effectors. It is also interesting to point out that despite extensive analysis of molecular events occurring in some LMMs, whether these deaths are due to PCD or are mechanistically equivalent to HR require further investigations. Indeed in many LMM, deeper biochemical or cytological studies are missing and further characterization would be required to classify LMM according to the different types of PCD involved in their phenotypes.

Altogether, proteins altered in LMM and suppressors of LMM, with other numerous factors, build an extremely complex network regulating the decision to die or not. It is also possible to assume the involvement of a core complex as a central hub integrating several PCD pathways leading to the cell suicide. Deeper genetic characterization, with the analysis of epistatic relationships between LMM and their suppressors will certainly help to decipher the links between the factors of this network and possible cross-talks between different pathways.

As mentioned several times, phytohormones such as SA, JA, and ETH have prominent roles in regulation of HR, and thus PCD, but also more generally in immune responses, senescence, abiotic stress response, plant growth and development (Rivas-San Vicente and Plasencia, 2011; Santino et al., 2013; Zhang and Zhou, 2013; Miura and Tada, 2014). Therefore, phytohormones involvement can be crucial in cell death, and LMM displaying constitutive or conditional activation in their signaling pathways are powerful tools which have also helped to highlight crosstalk between these pathways (Greenberg et al., 2000; Devadas et al., 2002; Derksen et al., 2013; Satoh et al., 2013).

Another open question is which cellular factors are involved in the execution of PCD itself. Indeed, no close orthologs of animal caspases were identified in plants, however, there is extensive evidence for the involvement of caspase-like activities during plant PCD (Reape and McCabe, 2010). To date, only two caspase-like proteins have been identified in plants via LMM approaches. Surprisingly, METACASPASE 1 and 2 (MC1 and MC2) appear to play antagonistic roles in the control of PCD (Coll et al., 2010). Further work will be required to elucidate the specific roles of these caspase-like activities at distinct stages during PCD, the significance of their subcellular localization, their substrate specificities, and the sequence in which they operate and interact with one another.

### Conflict of interest statement

The authors declare that the research was conducted in the absence of any commercial or financial relationships that could be construed as a potential conflict of interest.
